# BioGPS: an extensible and customizable portal for querying and organizing gene annotation resources

**DOI:** 10.1186/gb-2009-10-11-r130

**Published:** 2009-11-17

**Authors:** Chunlei Wu, Camilo Orozco, Jason Boyer, Marc Leglise, James Goodale, Serge Batalov, Christopher L Hodge, James Haase, Jeff Janes, Jon W Huss, Andrew I Su

**Affiliations:** 1 Genomics Institute of the Novartis Research Foundation, 10675 John Jay Hopkins Dr., San Diego, CA 92121, USA

## Abstract

Online gene annotation resources are indispensable for analysis of genomics data. However, the landscape of these online resources is highly fragmented, and scientists often visit dozens of these sites for each gene in a candidate gene list. Here, we introduce BioGPS http://biogps.gnf.org, a centralized gene portal for aggregating distributed gene annotation resources. Moreover, BioGPS embraces the principle of community intelligence, enabling any user to easily and directly contribute to the BioGPS platform.

## Abstract 2

BioGPS is a community based customisable gene annotation portal bringing together gene annotation resources on to a single platform.

## Rationale

In the past decade, many technology platforms have been developed that allow researchers to generate data on a genome scale. For example, profiling technologies have been developed for highly parallel measurements of gene expression, copy number, genotype, and epigenetic state. The data derived from these high-throughput approaches can then be used to generate new hypotheses or inferences of gene function. In contrast to experiments focusing on a specific gene or gene family, these genome-scale experiments typically result in the identification of a list of candidate genes that are relatively unfamiliar to any single researcher. Hit lists identified by these methods can often span many protein classes and signaling pathways. In many cases, these genes may also have little or no previous functional characterization.

Researchers are then faced with the daunting task of prioritizing these candidate genes for detailed functional and mechanistic studies. Dozens of gene annotation resources and model organism databases serve prominent roles in the genetics and genomics communities. Take, for example, the instance where a researcher has identified hundreds or even thousands of differentially expressed genes between a cancer sample and a matched control. In prioritizing this gene list, many researchers would commonly search Entrez Gene [1] and Ensembl [2] as a first stop for many descriptions of critical gene annotation information, including primary sequence data, genome position, associated Gene Ontology terms, gene structure, and genetic variation. Other researchers may then consult the Mouse Genome Database (MGD) [3] and Rat Genome Database (RGD) [4] for annotation focused on these model organisms, including knockout phenotypes and quantitative trait loci. Molecular and cellular biologists may then visit the STRING database for protein interaction data [5]. Other researchers may query reference Gene Atlas expression data using the SymAtlas web site [6]. In addition, there are a wide variety of gene annotation sites targeting more specific communities, including a database describing the targets of the transcription factor CREB [7], the Allen Brain Atlas showing high-resolution expression information by *in situ *hybridization in the mouse brain [8], and the TargetScan database for microRNA target prediction [9]. Hundreds of such online resources for mammalian gene annotation currently exist [10,11].

Although the wide breadth of available resources is clearly a benefit to the community, there is no single resource that completely describes everything that a researcher might want to know about a gene's function. Each gene annotation resource presents a particular slice of the available gene annotation, generally corresponding to the developers' view of what their users are interested in. Consequently, many researchers (and in particular researchers who are investigating candidate genes from genome-wide analyses) end up visiting many different sites for each gene of interest in order to get as complete a picture as possible of gene function.

This system is highly inefficient and cumbersome for end users. User interfaces vary dramatically, and researchers must learn and remember how to navigate each site. Each site often accepts a different set of gene identifiers (Entrez Gene, Ensembl, Refseq, Unigene, and so on), making it difficult for users to find their gene of interest. This problem is even more complicated in cases where the official HUGO Gene Nomenclature Committee (HGNC) gene symbol is not the most commonly used symbol in the literature (for example, *TP53 *and *P53*). Finally, new online resources are continually being developed, and staying abreast of these tools and evaluating their utility is a time-consuming and recurring task.

Moreover, this system is highly inefficient for web site developers. For example, every gene portal needs to implement at some level the basic functionality for searching for genes (for example, by symbol, identifier, location, sequence). Every gene portal also must make some effort at resolving synonyms that have been historically used in the literature. And every gene portal must also implement a mechanism for data updates from primary sources. Overall, a relatively large percentage of development effort duplicates existing but essential functionality that is common to all gene portals, and a relatively small percentage of effort is devoted to the innovative data and features of any specific gene portal.

Here, we introduce BioGPS, a gene annotation portal based on a loose federation of existing genetic and genomic resources. BioGPS allows users to easily explore the landscape of gene annotation resources for one or more genes of interest. BioGPS currently focuses on annotation for human, mouse, and rat genes. BioGPS also emphasizes two key design features. First, BioGPS is based on a simple, unstructured plugin interface that allows for simple community extensibility. Second, BioGPS also implements a powerful user interface that enables precise user customizability. In sum, these two design principles enable BioGPS to harness the principle of community intelligence toward the goal of efficiently organizing and querying online gene annotation resources.

## Basic gene portal functionality

Like many online gene annotation resources, BioGPS maintains a gene annotation database that combines information from public sources with data generated by our group. Scientists can find their gene or genes of interest using a flexible search interface, which accepts most public gene identifiers (Entrez Gene, Ensembl, Refseq, Uniprot, Affymetrix, and so on) and gene annotation identifiers (Gene Ontology, Interpro), as well as coordinates of a genomic interval. Selecting a gene out of the search result list displays a gene annotation report. In the case of BioGPS, the default gene annotation report focuses on our reference 'Gene Atlas' data sets, which show gene expression patterns from a diverse set of tissues and cell types [6,12,13].

However, our further development effort of BioGPS was driven by two key observations. First, not all users were primarily interested in our reference gene expression data, and second, not all gene annotation data that might be relevant to all users could be stored in a single database. Therefore, in addition to the default gene annotation report described above, BioGPS provides several other gene report 'layouts' that correspond to other common use cases. Each layout is an arrangement of 'plugins', and plugins primarily rely on third-party websites for content. For example, the 'Reagents' layout contains several plugins, each of which shows the molecular biology reagents available from a commonly used reagent provider. The 'Literature' layout focuses on literature searching, displaying plugins for both PubMed and Google Scholar. The 'Model Organism Databases' layout shows plugins for the MGD [3] and RGD [4] sites. The 'Wikipedia' layout shows the Gene Wiki plugin (which, like BioGPS, is another recent community intelligence initiative) [14]. The 'KEGG' layout shows biological pathways relevant to any particular gene of interest [15]. Finally, the 'Exon Atlas' layout displays a plugin showing data for a previously unpublished reference expression data set using a mouse exon array. Depending on which gene annotation resources are most relevant, a user can easily switch between layouts using a simple drop-down menu.

The layout system allows users to easily and directly view gene-centric data from multiple online data sources without having to initiate a query at each site. Although BioGPS often provides easy access to a site's basic 'gene report' page, drilling deeper into a given site still requires more specific knowledge of each site's unique navigation and search features.

The BioGPS framework is unique in its focus on aggregating distributed web content in a flexible layout system, a model that is highly amenable to extension and customization by external users and developers. Therefore, we next turned to the task of allowing users to directly extend BioGPS, both by adding additional third-party gene annotation resources, and by customizing additional layouts to suit their specific needs.

## BioGPS community extensibility via a simple, unstructured data format

BioGPS itself hosts a relatively limited amount of gene annotation data, and the majority of content is obtained through its role as a content aggregator of other online gene annotation resources. Unlike other efforts to synthesize context using highly structured formats (discussed in more detail below), BioGPS utilizes a virtually unstructured format for integrating disparate gene annotation resources. This data format is the simplest online format available - HyperText Markup Language, or HTML. The HTML format, of course, is the language by which the vast majority of web pages are displayed and is typically transferred using the common HyperText Transfer Protocol (HTTP).

In utilizing this lightweight data sharing model, BioGPS offers complementary strengths and weaknesses to more structured data exchange formats. For example, HTML intermixes the data itself with the instructions on presentation of that data. This property directly points to the limitation of this unstructured data model, that complex analyses cannot be constructed or chained together because the data do not have semantic context. However, the primary advantage of an unstructured HTML interface is that it is very easy to learn and implement. Learning to construct HTML using computer programs is among the most basic programming applications, and there exist many programming libraries that simplify this process. Of particular relevance to BioGPS, many interested graduate students and postdocs have used this level of computer programming skill to create simple web sites to display their experimentally generated data sets or bioinformatic predictions (for example, [7,16-18]), and it is this type of web site that has contributed to the explosion in the number of online resources. Moreover, HTML is completely flexible with regard to presentation, allowing the display of images and free text as easily as tables and genomic coordinates.

**Figure 1:**
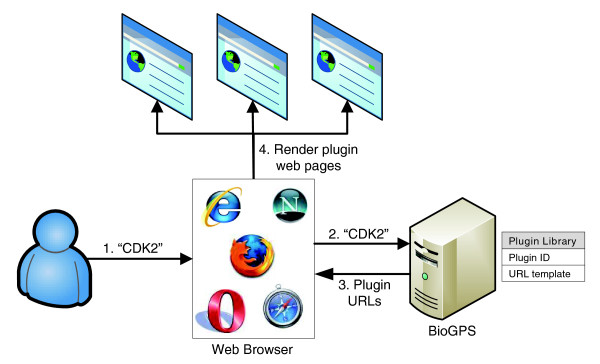
Schematic representation of BioGPS. In step 1, the user loads the BioGPS site in a web browser and inputs a query (here, 'CDK2'). In step 2, the web browser transmits the query to the BioGPS server. In step 3, BioGPS resolves the query into a gene entity, and returns fully formed URLs for plugins in the plugin library. In step 4, the user's web browser retrieves content from the plugin providers for the gene of interest and renders it within the customizable BioGPS layout.

Using simple HTML interfaces, BioGPS interacts with users and third-party plugin providers according to the outline in Figure 1. BioGPS maintains a plugin library that stores a 'Uniform Resource Locator (URL) template' for each registered plugin. This URL template indicates the basic syntax by which the external plugin server can be accessed and the type of gene identifier (for example, Entrez Gene, Ensembl, RefSeq) the plugin server will accept. When a user then queries BioGPS for a particular gene of interest, BioGPS resolves the user query to a canonical gene entity, translates that gene into all possible external identifiers, and then converts each plugin URL template into an actual website address. Those web site addresses are then passed back to be rendered in the user's browser as detailed in the next section. This design mirrors a traditional star schema in database warehouse design, where BioGPS serves as the fact table and where the dimension tables are distributed across many third-party servers [19]. This simple HTML-based data sharing mechanism allows most existing online tools to be easily integrated as BioGPS plugins, and it also allows new resources to be packaged as BioGPS plugins with minimal additional effort.

**Figure 2:**
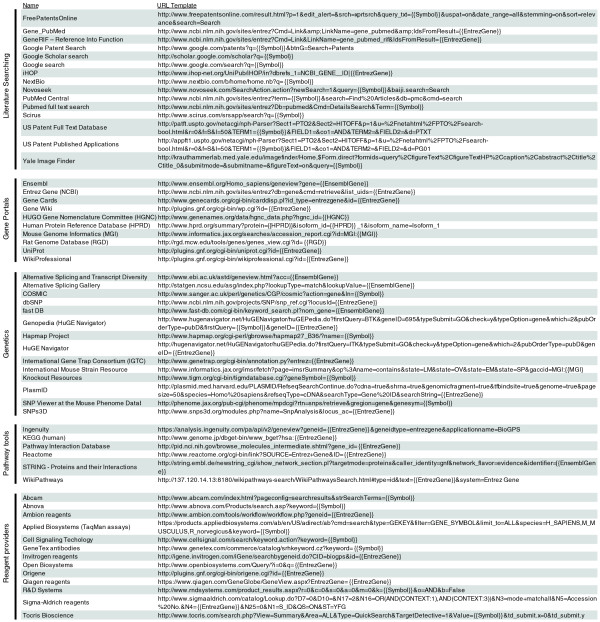
A partial list of BioGPS plugins. Valid URLs can be formed by substituting gene-specific variables in the URL templates above. For example, for the gene *CDK2*, {{Symbol}} = CDK2, {{EntrezGene}} = 1017 (or 12566 for mouse), {{EnsemblGene}} = ENSG00000123374, {{HGNC}} = 1771, {{HPRD}} = 00310, {{MGI}} = 104772, and {{RGD}} = 70486. Plugin URL templates can also utilize variables for Unigene ID, RefSeq transcript or protein IDs, Protein Data Bank (PDB) ID, and Online Mendelian Inheritance in Man (OMIM) accession.

The BioGPS plugin library currently has over 150 registered plugins (partial listing in Figure 2). The selection of plugins spans many different areas and resources, including literature searching, model organism databases, genetics resources, pathways tools, and reagent providers. These online resources span over 65 unique domain names, indicating the relative ease with which existing resources can be registered as BioGPS plugins. In addition, BioGPS hosts a gene expression plugin that displays reference expression patterns from the Gene Atlas data sets [6] and expression quantitative trait loci studies [20], as well as new data sets for an updated mouse Gene Atlas [GEO:GSE10246] [12] and exon array atlas [GEO:GSE15998].

Importantly, any user who has created an optional BioGPS user account can also register new plugins in the plugin library. This feature enables the entire community of scientists to directly extend BioGPS by registering existing sites as BioGPS plugins. Because of its simplicity, the HTML-based plugin interface is easy enough for many web-savvy users to understand and utilize, even if they themselves are not website developers. Moreover, integration with BioGPS offers many advantages to external developers of new applications. Developers can completely delegate responsibility to BioGPS for both gene synonym resolution and mappings between public identifiers. These developers also immediately gain access to the substantial BioGPS user base. For these and related reasons, we believe that BioGPS will also encourage the creation of online resources by reducing the overhead and duplication of functionality.

## BioGPS user customizability using layouts

Since BioGPS provides a simple mechanism by which online gene annotation resources can easily be registered as plugins, the BioGPS user interface is critical for its utility to users. Clearly not all 150+ plugins will be relevant to every user and every use case, and certainly any interface that attempted to display all plugin content at once would be unusable. Therefore, BioGPS's second key design principle is user customizability.

BioGPS utilizes a technically simple user interface. Plugins are rendered in IFRAMEs, which are essentially web browsers embedded within a main web browser. In this way, BioGPS is truly a federated hub, providing users' browsers with deep-links into plugin servers based on the URL template. Plugin content is returned and rendered using simple HTML.

**Figure 3:**
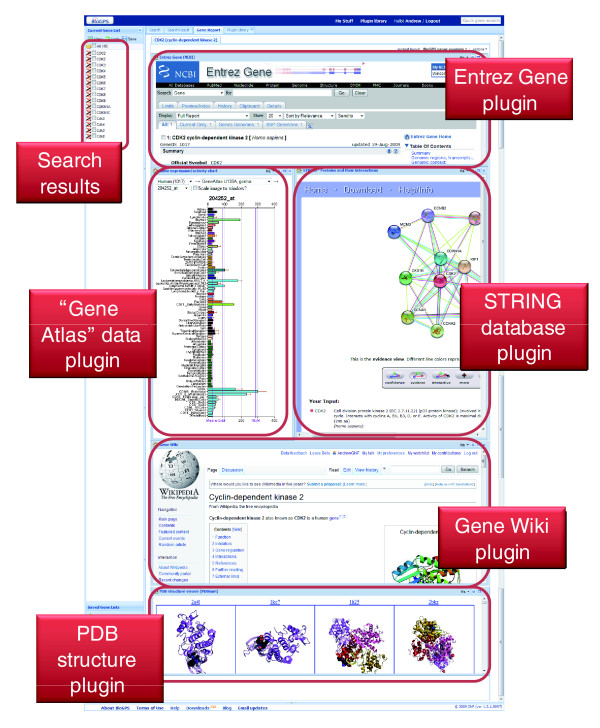
Screenshot of a custom BioGPS gene report layout for the gene *CDK2*. Registered users can easily create custom layouts using any plugins in the plugin library. Each plugin is rendered within its own window that can be moved, resized, and maximized using standard controls.

As both scientists and developers, we recognize that different users are interested in learning about different aspects of gene function. Previously, we described the pre-defined layouts offered in BioGPS that correspond to common use cases. In addition, users who register for an optional user account are able to personalize and save additional gene report layouts. These custom layouts enable each user to tailor BioGPS to their individual interests using any of the registered plugins in the plugin library (an example custom layout is shown in Figure 3). Within a layout, plugins are visualized using a windowing framework in which, similar to common operating systems, windows can be repositioned and resized using standard click-and-drag mouse actions. Switching between layouts (either pre-defined or personalized) can be done using a simple drop-down menu.

## Community intelligence in science

If the only distinguishing features in BioGPS were an extensible plugin framework and a customizable user interface, BioGPS would be a useful tool with incremental advantages relative to existing online resources. However, we believe that BioGPS has significant additional potential because it leverages those design features to harness the principle of community intelligence.

Community intelligence initiatives are based on the idea that a large community of users can collectively and collaboratively synthesize knowledge. The most visible example of this idea is the online encyclopedia Wikipedia, in which over nine million registered users have collaboratively written almost three million articles. Community intelligence specifically targets contributions from the 'Long Tail', the large population of users who make individually small (but collectively large) contributions of content. More recently, web-based community intelligence efforts have been applied toward scientific goals, and specifically toward the goal of comprehensive genome-wide gene annotation. Recent examples include the Gene Wiki [14], WikiProteins [21], WikiGenes [22], and WikiPathways [23].

Although these previous efforts address a different scientific goal, many of the same principles are required for success. Specifically, community intelligence applications must initiate a positive feedback loop involving three components: scientific utility, community usage, and community contributions. In the absence of this positive feedback loop, these community intelligence applications never achieve the critical mass of users and activity on which they depend.

Regarding the first step of this cycle, quantitative metrics for scientific utility of BioGPS are difficult to measure. However, we have seeded the BioGPS plugin library with many commonly used gene annotation resources, establishing a baseline level of utility to scientists. Moreover, we have included several reference datasets that have been extensively utilized in the microarray community through our SymAtlas website [6]. Finally, we offer users several default layouts corresponding to common scientific use cases. We believe that these basic features offer a solid foundation of scientific utility.

The second step in this positive feedback cycle is establishing a critical mass of community usage. BioGPS was publicly launched in August 2008. Although more than 90% of BioGPS visitors use the site without logging in, there are also over 1,500 users who have registered for BioGPS accounts, which enable advanced features. Of these registered users, 128 have logged in 5 or more times. Traffic to the website has steadily grown, and BioGPS currently receives over 130,000 page hits from almost 10,000 unique visitors per month (only counting traffic from outside our institute). While we believe there is still plenty of growth potential, these usage statistics indicate a broad and consistent user base on which this community intelligence initiative can build.

Finally, the third step in this positive feedback loop is establishing community contributions. Direct contributions from users can currently come in three forms. First, BioGPS users can add custom plugins to the plugin library. Because accessing gene annotation resources through BioGPS offers tangible and potentially significant benefits, we believe that scientists will be motivated to register online resources as BioGPS plugins. Second, and perhaps even more importantly, we believe that developers who do not have either the skills or the motivation to create a full online gene annotation application may see value in creating a BioGPS plugin for their data. By handling many of the mundane tasks of gene resource development and maintenance, BioGPS allows developers to focus on the novel and innovative aspects of their data. So far, the combination of these two mechanisms has resulted in over 50 plugins registered by external BioGPS members. Third, BioGPS users contribute to the pool of community intelligence simply by creating custom gene report layouts. BioGPS uses all user-created custom layouts to calculate aggregated plugin usage data. BioGPS can then assess and report the popularity (and, by inference, the utility) of each plugin, thereby providing a very efficient marketplace of online resources. In the same way that the Google search engine uses links to a given web page as a measure of utility, BioGPS uses plugin usage in layouts as an approximation of scientific utility to the biological community. Because these metrics of utility can be easily computed from aggregated usage patterns, BioGPS users are also contributing to the BioGPS community intelligence simply by using this provided functionality. So far, registered users have created 1,100 custom gene report layouts, and over one-third of registered users have created at least one custom gene report layout. Based on current usage, the most popular plugins are shown in Additional data file 1, and up-to-date rankings of plugin popularity can always be viewed in the BioGPS plugin library.

In short, the community intelligence positive feedback loop begins with a baseline level of scientific utility that then attracts users, a set of users that is then encouraged and enabled to become contributors, and a population of contributors and contributions that then increases the level of scientific utility. We believe that the usage patterns above indicate that BioGPS has and will continue to successfully leverage community intelligence.

## The landscape of gene annotation resources

As mentioned previously, hundreds of gene annotation databases are currently available. Although the majority of these resources operate as silos of data and functionality, other bioinformatics scientists have previously recognized the potential benefits of integration and proposed potential solutions. Thus far, the bioinformatics community has primarily focused on structured data interfaces. In particular, the Distributed Annotation System (DAS) is a data exchange protocol created by a consortium of bioinformatics developers aimed at enabling sharing and transferring of gene annotation information [24]. DAS is widely used by model organism databases and genome browsers for exchanging and integrating annotation information referenced by genomic coordinates. It is based on a web services protocol and an Extensible Markup Language (XML) data format. A structured data exchange format like DAS has many advantages, most notably enabling more complex analysis, visualization, and integration based on semantic awareness of the data types.

Relative to the BioGPS data exchange format described above, DAS is a highly structured data format in which each piece of data is strongly typed. A structured data exchange format has many advantages, most notably the enabling of more complex analyses and visualizations based on semantic awareness of the data meaning. DAS completely separates the data from the presentation and analysis of that data. However, serving and consuming structured data also requires a relatively high degree of bioinformatics and programming sophistication. For example, the main DAS specifications are over 8,000 words long [25,26], and perhaps as a result, over 70% of human, mouse, and rat DAS services registered at dasregistry.org are hosted by just three organizations. Furthermore, another potential disadvantage of a structured data format is that data types not defined in the specification cannot be natively shared or visualized.

We introduce BioGPS as an easily extensible and customizable gene portal. Utilizing a simple HTML-based plugin interface, BioGPS enables users to easily aggregate data on a gene by gene basis from more than 150 external sources, and to personalize their gene report using BioGPS layouts. Moreover, BioGPS enables the entire community to register new plugins, increasing the breadth and depth of accessible gene annotation and allowing external developers to take advantage of the BioGPS user base and core searching functionality. In contrast to other catalogs of online gene annotation resources [10,11], the BioGPS plugin library additionally allows users to rank plugins by utility, as measured by the entire community's usage of each plugin in BioGPS layouts. Although BioGPS superficially is a simple gene annotation hub, we believe it is unique for leveraging the idea of community intelligence toward collaboratively building an online gene annotation resource.

It is important to note that BioGPS is not a substitute for existing gene portals, nor does the simple HTML interface used by BioGPS replace efforts like DAS for structured data transfer. In fact, we look forward to the future when all gene annotation resources are connected by semantically aware web services and Resource Description Framework (RDF) triplestores [27]. Nevertheless, we believe that the simple data models and integration strategies presented here will engage a larger community of scientists in this community intelligence effort, and that BioGPS will play an important role in integrating current and future Web 1.0 resources.

BioGPS is openly and freely accessible online. Users can optionally register for a user account to enable customization features, and registration is also completely free. Screencast tutorials can be found in the Help section.

## Software architecture

BioGPS was built following a three-tiered application model. The data tier was implemented using Oracle version 11.1.0.7, and data tables were populated by importing gene annotations and relationships from common gene annotation databases (predominantly NCBI and Ensembl). The business logic tier was built using C# and the .NET Framework version 3.5, utilizing the Windows Communication Foundation (WCF) to expose Representational State Transfer (REST)-based web services for querying and data retrieval. The presentation layer, which includes the plugin and layout management system, was built using the Django web framework and extensively uses the EXTJS JavaScript library.

## Abbreviations

DAS: Distributed Annotation System; HGNC: HUGO Gene Nomenclature Committee; HTML: HyperText Markup Language; MGD: Mouse Genome Database; RDF: Resource Description Framework; RGD: Rat Genome Database; URL: Uniform Resource Locator.

## Authors' contributions

AIS conceived of the project, and CW and CO designed key aspects of BioGPS. CW, CO, JB, ML, JG, SB, CLH, JH, JJ, and JWH implemented the core platform and key plugins. AIS wrote the manuscript. All authors read and approved the final manuscript.

## Additional data files

The following additional data are available with the online version of this paper: a table of current popularity scores for BioGPS plugins as measured by usage in custom layouts (Additional data file 1).

## Supplementary Material

Additional data file 1 Up to date popularity scores are always available online in the BioGPS plugin library. Click here for file

## References

[B1] Maglott D Ostell J Pruitt KD Tatusova T Entrez Gene: gene-centered information at NCBI. Nucleic Acids Res 2007 35 D26 31 10.1093/nar/gkl993 17148475 PMC1761442

[B2] Flicek P Aken BL Beal K Ballester B Caccamo M Chen Y Clarke L Coates G Cunningham F Cutts T Down T Dyer SC Eyre T Fitzgerald S Fernandez-Banet J Graf S Haider S Hammond M Holland R Howe KL Howe K Johnson N Jenkinson A Kahari A Keefe D Kokocinski F Kulesha E Lawson D Longden I Megy K Ensembl 2008. Nucleic Acids Res 2008 36 D707 714 10.1093/nar/gkm988 18000006 PMC2238821

[B3] Bult CJ Eppig JT Kadin JA Richardson JE Blake JA The Mouse Genome Database (MGD): mouse biology and model systems. Nucleic Acids Res 2008 36 D724 728 10.1093/nar/gkm961 18158299 PMC2238849

[B4] Twigger SN Shimoyama M Bromberg S Kwitek AE Jacob HJ The Rat Genome Database, update 2007 - easing the path from disease to data and back again. Nucleic Acids Res 2007 35 D658 662 10.1093/nar/gkl988 17151068 PMC1761441

[B5] Jensen LJ Kuhn M Stark M Chaffron S Creevey C Muller J Doerks T Julien P Roth A Simonovic M Bork P von Mering C STRING 8 - a global view on proteins and their functional interactions in 630 organisms. Nucleic Acids Res 2009 37 D412 416 10.1093/nar/gkn760 18940858 PMC2686466

[B6] Su AI Wiltshire T Batalov S Lapp H Ching KA Block D Zhang J Soden R Hayakawa M Kreiman G Cooke MP Walker JR Hogenesch JB A gene atlas of the mouse and human protein-encoding transcriptomes. Proc Natl Acad Sci USA 2004 101 6062 6067 10.1073/pnas.0400782101 15075390 PMC395923

[B7] Zhang X Odom DT Koo SH Conkright MD Canettieri G Best J Chen H Jenner R Herbolsheimer E Jacobsen E Kadam S Ecker JR Emerson B Hogenesch JB Unterman T Young RA Montminy M Genome-wide analysis of cAMP-response element binding protein occupancy, phosphorylation, and target gene activation in human tissues. Proc Natl Acad Sci USA 2005 102 4459 4464 10.1073/pnas.0501076102 15753290 PMC555478

[B8] Lein ES Hawrylycz MJ Ao N Ayres M Bensinger A Bernard A Boe AF Boguski MS Brockway KS Byrnes EJ Chen L Chen L Chen TM Chin MC Chong J Crook BE Czaplinska A Dang CN Datta S Dee NR Desaki AL Desta T Diep E Dolbeare TA Donelan MJ Dong HW Dougherty JG Duncan BJ Ebbert AJ Eichele G Genome-wide atlas of gene expression in the adult mouse brain. Nature 2007 445 168 176 10.1038/nature05453 17151600

[B9] Friedman RC Farh KK Burge CB Bartel DP Most mammalian mRNAs are conserved targets of microRNAs. Genome Res 2009 19 92 105 10.1101/gr.082701.108 18955434 PMC2612969

[B10] Galperin MY Cochrane GR Nucleic Acids Research annual Database Issue and the NAR online Molecular Biology Database Collection in 2009. Nucleic Acids Res 2009 37 D1 4 10.1093/nar/gkn942 19033364 PMC2686608

[B11] Brazas MD Fox JA Brown T McMillan S Ouellette BF Keeping pace with the data: 2008 update on the Bioinformatics Links Directory. Nucleic Acids Res 2008 36 W2 4 10.1093/nar/gkn399 18586831 PMC2447757

[B12] Lattin JE Schroder K Su AI Walker JR Zhang J Wiltshire T Saijo K Glass CK Hume DA Kellie S Sweet MJ Expression analysis of G protein-coupled receptors in mouse macrophages. Immunome Res 2008 4 5 10.1186/1745-7580-4-5 18442421 PMC2394514

[B13] Walker JR Su AI Self DW Hogenesch JB Lapp H Maier R Hoyer D Bilbe G Applications of a rat multiple tissue gene expression data set. Genome Res 2004 14 742 749 10.1101/gr.2161804 15060018 PMC383321

[B14] Huss JW Orozco C Goodale J Wu C Batalov S Vickers TJ Valafar F Su AI A gene wiki for community annotation of gene function. PLoS Biol 2008 6 e175 10.1371/journal.pbio.0060175 18613750 PMC2443188

[B15] Kanehisa M Araki M Goto S Hattori M Hirakawa M Itoh M Katayama T Kawashima S Okuda S Tokimatsu T Yamanishi Y KEGG for linking genomes to life and the environment. Nucleic Acids Res 2008 36 D480 484 10.1093/nar/gkm882 18077471 PMC2238879

[B16] Dix MM Simon GM Cravatt BF Global mapping of the topography and magnitude of proteolytic events in apoptosis. Cell 2008 134 679 691 10.1016/j.cell.2008.06.038 18724940 PMC2597167

[B17] Xu S McCusker J Krauthammer M Yale Image Finder (YIF): a new search engine for retrieving biomedical images. Bioinformatics 2008 24 1968 1970 10.1093/bioinformatics/btn340 18614584 PMC2732221

[B18] Yue P Melamud E Moult J SNPs3D: candidate gene and SNP selection for association studies. BMC Bioinformatics 2006 7 166 10.1186/1471-2105-7-166 16551372 PMC1435944

[B19] Wang L Zhang A Ramanathan M BioStar models of clinical and genomic data for biomedical data warehouse design. Int J Bioinform Res Appl 2005 1 63 80 10.1504/IJBRA.2005.006903 18048122 PMC2574433

[B20] Wu C Delano DL Mitro N Su SV Janes J McClurg P Batalov S Welch GL Zhang J Orth AP Walker JR Glynne RJ Cooke MP Takahashi JS Shimomura K Kohsaka A Bass J Saez E Wiltshire T Su AI Gene set enrichment in eQTL data identifies novel annotations and pathway regulators. PLoS Genet 2008 4 e1000070 10.1371/journal.pgen.1000070 18464898 PMC2346558

[B21] Mons B Ashburner M Chichester C van Mulligen E Weeber M den Dunnen J van Ommen GJ Musen M Cockerill M Hermjakob H Mons A Packer A Pacheco R Lewis S Berkeley A Melton W Barris N Wales J Meijssen G Moeller E Roes PJ Borner K Bairoch A Calling on a million minds for community annotation in WikiProteins. Genome Biol 2008 9 R89 10.1186/gb-2008-9-5-r89 18507872 PMC2441475

[B22] Hoffmann R A wiki for the life sciences where authorship matters. Nat Genet 2008 40 1047 1051 10.1038/ng.f.217 18728691

[B23] Pico AR Kelder T van Iersel MP Hanspers K Conklin BR Evelo C WikiPathways: pathway editing for the people. PLoS Biol 2008 6 e184 10.1371/journal.pbio.0060184 18651794 PMC2475545

[B24] Jenkinson AM Albrecht M Birney E Blankenburg H Down T Finn RD Hermjakob H Hubbard TJ Jimenez RC Jones P Kahari A Kulesha E Macias JR Reeves GA Prlic A Integrating biological data - the Distributed Annotation System. BMC Bioinformatics 2008 9 Suppl 8 S3 10.1186/1471-2105-9-S8-S3 18673527 PMC2500094

[B25] DAS2 Protocol http://biodas.org/documents/das2/das2_protocol.html

[B26] Retrieving DAS2 genomic sequence and annotation feature records http://biodas.org/documents/das2/das2_get.html

[B27] Goble C Stevens R State of the nation in data integration for bioinformatics. J Biomed Inform 2008 41 687 693 10.1016/j.jbi.2008.01.008 18358788

